# Anatomical variations of the deep femoral artery: a dissection-based study

**DOI:** 10.1007/s00276-025-03702-z

**Published:** 2025-09-15

**Authors:** Elsje Louise de Villiers, Kerri Keet

**Affiliations:** Division of Clinical Anatomy, Department of Biomedical Sciences, Faculty of Medicine and Health Sciences, Stellenbosch University, Stellenbosch, South Africa

**Keywords:** Deep femoral artery, Medial circumflex femoral artery, Lateral circumflex femoral artery, Anatomical variation, Femoral artery

## Abstract

**Purpose:**

The deep femoral artery and its branches may vary in origin, position and diameter. Variations in these vessels may have clinical implications for interventional procedures; however, there is a shortage of published information from South African studies. Therefore, this study investigated the variations of the femoral artery (FA), deep femoral artery (DFA), medial circumflex femoral artery (MCFA), and lateral circumflex femoral artery (LCFA) in a dissection study, comparing findings between sides of the body and sexes.

**Methods:**

Following ethical approval, sixty lower limbs (20 Males and 10 females) were dissected to ascertain the site of DFA origin and the vessels from which the MCFA and LCFA originated. The distances between the inguinal ligament and the DFA origin, and between the DFA origin and the MCFA and LCFA origins were measured. External diameters of the FA, DFA, MCFA, and LCFA were determined. The findings were statistically compared between sides and sexes.

**Results:**

The DFA originated predominantly from the posterior (58.3%) and posterolateral (28.3%) aspects of the FA. The DFA was the primary origin for the MCFA (63.3%) and LCFA (75.0%), with a significant difference between sexes in the prevalence of the MCFA origin. Males exhibited a higher prevalence of low DFA origin (36.7%) than females (8.3%), whereas no significant differences were observed in the measurements between sides or sexes.

**Conclusion:**

The study revealed variations in the origins, distances, and diameters of the FA, DFA, MCFA, and LCFA. Awareness of these variations is crucial for interventional radiologists and orthopaedic surgeons to mitigate risks of iatrogenic injuries during procedures in the femoral region.

**Supplementary Information:**

The online version contains supplementary material available at 10.1007/s00276-025-03702-z.

## Introduction

The deep femoral artery (DFA), also known as the *profunda femoris*, is the largest branch of the femoral artery (FA) and is responsible for providing the primary Blood supply to the thigh. The FA, a continuation of the external iliac artery, enters the femoral triangle deep to the inguinal ligament. Approximately 20–60 mm below this ligament, the FA gives rise to the DFA. The DFA subsequently branches into the MCFA, LCFA, and three perforating branches, ultimately terminating as a fourth branch that connects with the popliteal artery. Variations in these arteries may involve differing origins and diameters, varying distances to key clinical landmarks such as the midpoint of the inguinal ligament (MIP), and instances of absence and duplication [25, 28].

Vascular variations such as absence, duplication or vessel hypoplasia, are suggested to result from an interplay of genes, environmental factors, and changes in regulatory pathways during embryological development. The arterial supply to the lower limb develops from two branches of the umbilical artery at 35–37 days of development. The first vessel to enter the developing lower limb is the axial (sciatic) artery, which later remodels and regresses. The second vessel forms the femoral artery and its branches after extensive vascular remodeling. Vascular remodeling is a complex process with vascular endothelial growth factor an important growth factor influencing the anatomy of the persistent vessels [21].

As the femoral artery and its branches are cannulated in interventional procedures, variations in the origin, position, and size of these vessels may have clinical implications. Due to the proximity of vessels and nerves within the femoral triangle, a high origin of the DFA may complicate procedures involving femoral artery puncture [13]. In such cases, the DFA may be inadvertently punctured instead of the FA, potentially resulting in the formation of arteriovenous fistulas and pseudoaneurysms [13]. Furthermore, a comprehensive understanding of the prevalence of the variant origins and the range of diameter of the LCFA is crucial during reconstructive surgeries involving anterolateral thigh (ALT) flaps to ensure adequate blood flow to the flap and to minimise the risk of flap necrosis [28]. Additionally, differences in the anatomy of the MCFA are important in trochanteric and intertrochanteric osteotomy procedures, since the MCFA is the main blood source for the head and neck of the femur. Knowledge of the variations of the MCFA may thus reduce the risk of intraoperative injuries and prevent vascular necrosis of the femoral head. Therefore, awareness of the variations of the DFA, MCFA, and LCFA may enhance procedural safety, potentially leading to improved patient outcomes and a reduced risk of iatrogenic injuries during plastic, reconstructive, or vascular procedures in the femoral and hip regions [28]. Furthermore, understanding differences in these structures between sexes and sides is essential for tailoring surgical approaches to individual patients, thereby optimising clinical outcomes and minimising complications [13, 16]. However, little research has been undertaken comparing variations of these vessels between sides and sexes.

This study therefore investigated the prevalence of variations and the range of dimensions of the FA, DFA, MCFA, LCFA and compared the findings between right and left lower limbs and between male and female embalmed body donors from a South African sample. The specific objectives included determining the site of DFA origin, identifying the vessels from which MCFA and LCFA arose, and measuring the external diameters, and the distances between anatomical points and these vessels.

## Materials and methods

This study was descriptive, dissection-based, cross-sectional, and carried out over ten months (January 2024–October 2024). A total of 40 bodies were assessed for inclusion. All of the bodies were embalmed in a 10.0% formalin solution and sourced from the body donation program of the institution. All donors were aged 18 years or older and both biological sexes were included, with both left and right lower limbs present. The exclusion criteria comprised bodies with tumours, surgical scars, trauma, or damage from previous dissection in the anterior and medial thigh regions. In addition, bodies with excessive desiccation or lower limb amputations were excluded.

### Data collection

The anterior thigh was previously preliminary dissected by health sciences students using *Gray’s Clinical Photographic Dissector* (Chapter 17, pp. 284–298) [10]. The DFA and its branches, the MCFA and LCFA were subsequently further exposed by the principal investigator by cleaning and dissecting the deep fascia with a standard dissection kit. The sartorius muscle was reflected from the anterior superior iliac spine when necessary for better access to the femoral vessels. The femoral sheath, containing the femoral artery and vein, was incised. The femoral vein was removed if required to expose the MCFA. The origin of the DFA from the FA was documented based on its location (posterior, lateral, etc.) [6]. The FA was divided into quadrants by two perpendicular lines to demarcate the anterior–posterior, and the medial–lateral aspects. If the DFA originated between these lines, it was classified as anterolateral, anteromedial, posterolateral, or posteromedial, respectively. The vessel from which the MCFA and LCFA originated was also recorded. To quantify the level of origin of the DFA, the distance between the origin of this vessel and the midpoint of the inguinal ligament was measured in a straight line. The midpoint (MIP) of the inguinal ligament was established as the halfway point between two pins placed on the pubic tubercle and ASIS respectively. The level of origin was classified as high (1-20 mm distal to MIP), middle (21-50 mm distal to MIP), and low (51-100 mm). In addition, straight line distances between the DFA origin and both the MCFA and the LCFA origins were measured. Finally, the external diameters of the FA (proximal to the origin of the DFA), and the DFA, MCFA, and the LCFA were measured at the origin of each vessel (Supplement 1; Figs. S1–S3). Measurements were taken using an ORIGIN digital calliper (0-150 mm). Each dimension was recorded three times, and vessel diameters were averaged from six measurements (three for anteroposterior and three for transverse distances).

### Data analysis

The Statistical Package for the Social Sciences (SPSS, version 29) was used in consultation with a biostatistician. The following statistical tests were performed with *p* < *0.05* considered as significant: Chi-square or Fisher exact tests to assess differences in prevalence between sides and sexes; Shapiro–Wilk Normality test to evaluate the distribution of the measurements; Student’s t-test or Mann–Whitney U test for comparing measurements between sexes; and paired t-test or Wilcoxon Signed Rank test for comparison between left and right sides. Bland–Altman reliability tests were performed on a 17.0% subsample to assess intra- and inter-observer reliability [3]. An anatomist at the same institution repeated the measurements after training was provided.

### Ethical considerations

Ethical approval was obtained from the institution (U24/03/311). Approval to include the body donors was obtained from the Health Officer under the National Health Act (Act 61 of 2003) of South Africa. Consent was obtained from the body donors prior to death or from the next-of-kin after death. The body donation programme follows the International Federation of Associations of Anatomists’s guidelines for body donation programmes.

This study was conducted in accordance with the ethical guidelines of the World Medical Association Declaration of Helsinki for medical research involving human participants.

## Results

Of the 40 bodies assessed, ten (20 lower limbs) were excluded, resulting in a final sample of 30 bodies (60 lower limbs, 30 per side), of which 20 were Male and 10 were female.

### Prevalence of variation

**Fig. 1:**
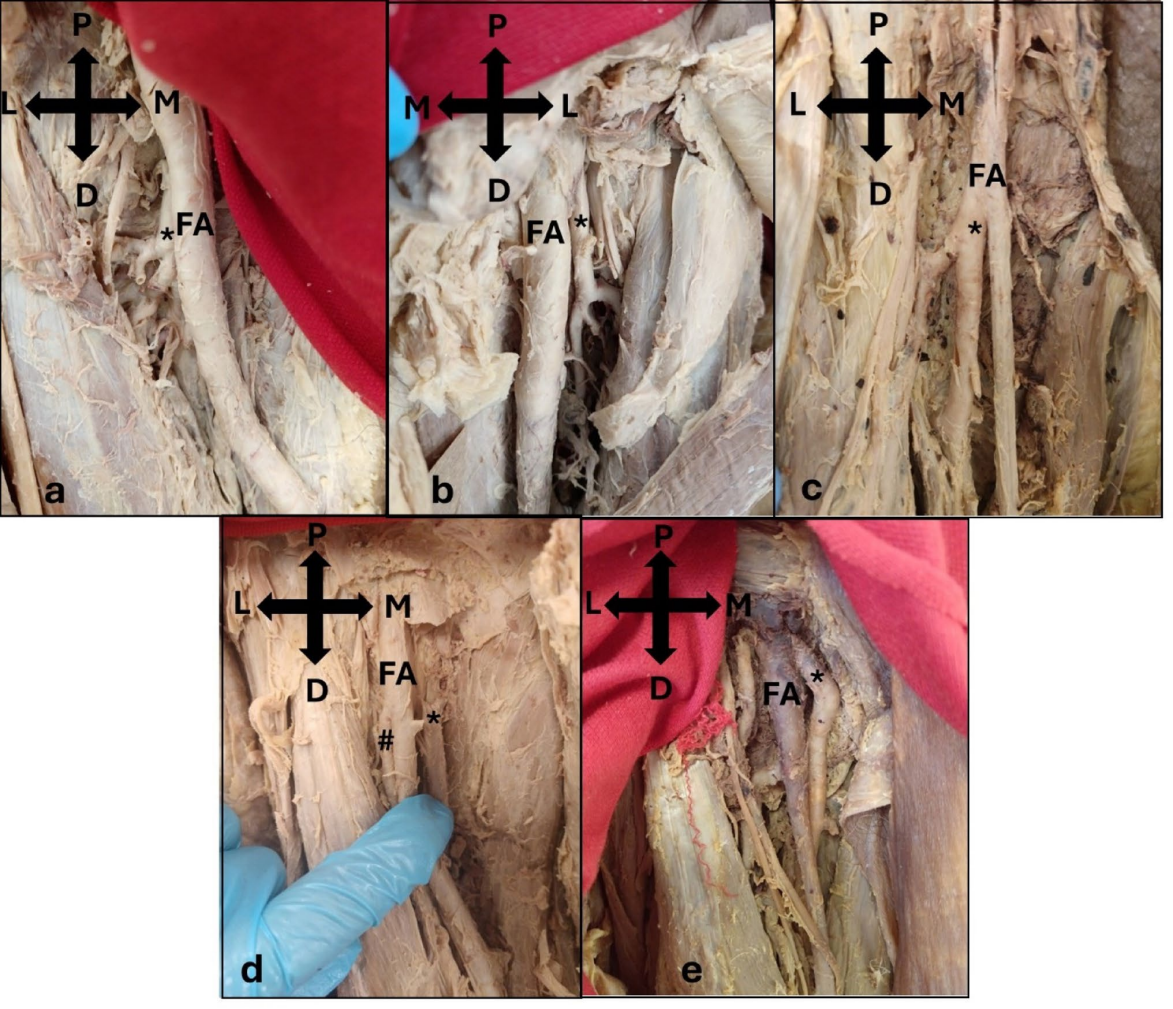
Photographs of the sites of the deep femoral artery (*) origin from the femoral artery (FA) including the **a** posterior origin (Right; Male); **b** posterolateral origin (Left; Male); **c** lateral origin (Right; Male); **d** posteromedial origin (Right; Female; deep femoral artery cut); and the **e** medial origin (Right; Female). P, Proximal; D, Distal; M, Medial; L, Lateral; #, lateral circumflex femoral artery

No instances were observed in which the DFA, MCFA, or LCFA were absent; thus, all vessels were present in all 60 lower limbs examined. The site of origin of the DFA varied between the posterior (58.3%), posterolateral (28.3%), lateral (10.0%), posteromedial (1.7%), and medial (1.7%) aspects of the FA, with no significant differences between the sides (p = 0.277) or sexes (p = 0.113, Table 1). However, the posterior site of origin was more common on the left side (31.6%) than on the right (26.7%) and more common in males (38.3%) than in females (20.0%). The posterolateral site of origin was similar in prevalence between the left (13.3%) and right (15.0%) sides, and more common in males (18.3%) than females (10.0%). In contrast, the lateral origin was more prevalent on the right side (8.3%) than the left (1.7%), and was only observed within males (10.0%). Finally, there was only one lower limb with the posteromedial (1.7%) and medial (1.7%) sites of origin respectively, both of which were on the left side and in females (Fig. 1).

**Table 1:** Prevalence of the variation in the site of origin of the deep femoral artery from the femoral artery according to side and sex

Site of Origin	Total number of limbs (%)	Side		Sex	
		Left Number of limbs (%)	Right Number of limbs (%)	Male Number of limbs (%)	Female Number of limbs (%)
Posterior	35 (58.3)	19 (31.6)	16 (26.7)	23 (38.3)	12 (20.0)
Posterolateral	17 (28.3)	8 (13.3)	9 (15.0)	11 (18.3)	6 (10.0)
Lateral	6 (10.0)	1 (1.7)	5 (8.3)	6 (10.0)	0 (0.0)
Posteromedial	1 (1.7)	1 (1.7)	0 (0.0)	0 (0.0)	1 (1.7)
Medial	1 (1.7)	1 (1.7)	0 (0.0)	0 (0.0)	1 (1.7)
*p-value*		*0.277*		*0.113*

**Fig. 2:**
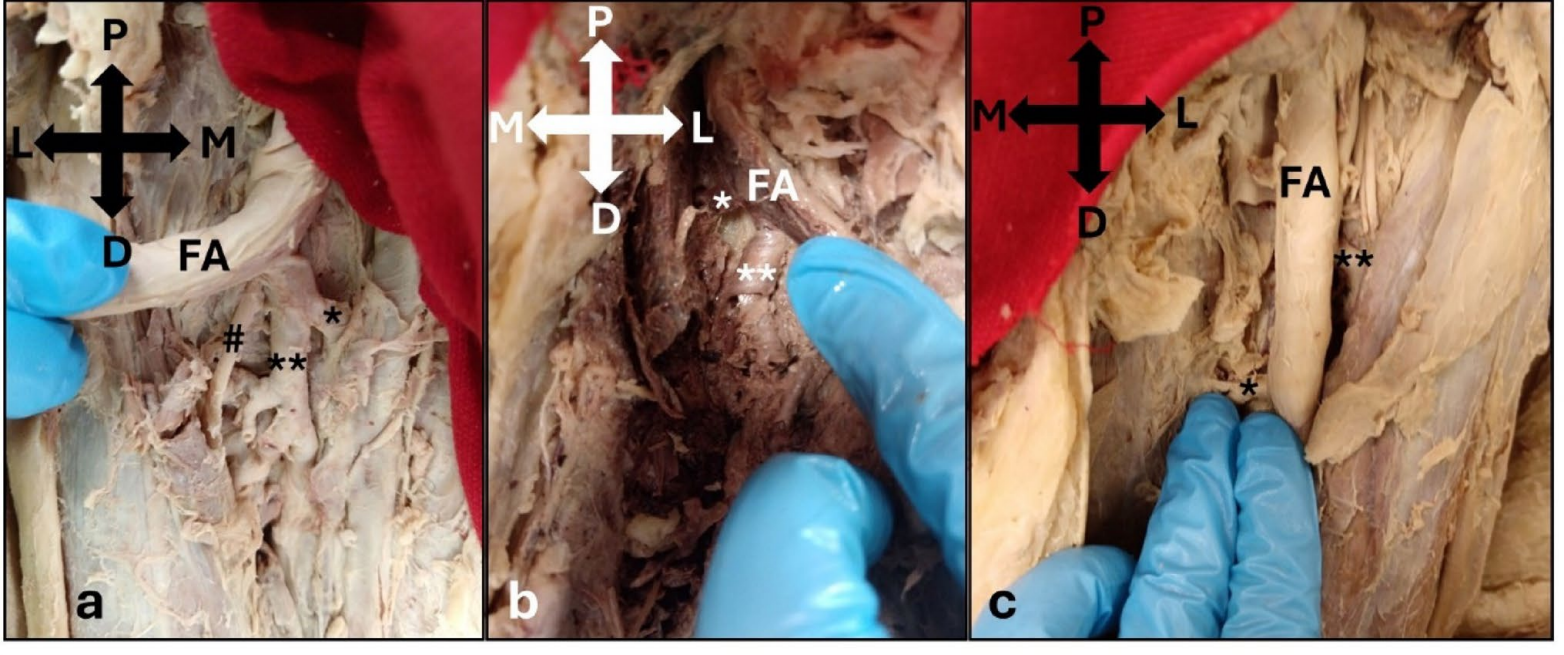
Photographs showing the variation in the origin of the medial circumflex femoral artery (*) including the **a** origin from the deep femoral artery (**) (Right; Male); **b** origin from the femoral artery proximal to the deep femoral artery (**) (Left; Female); and **c** origin from the femoral artery (FA) distal to the deep femoral artery (**) (Left; Male). P, Proximal; D, Distal; M, Medial; L, Lateral; #, lateral circumflex femoral artery

The MCFA originated from either the DFA (63.3%), the FA proximal to the DFA (30.0%), shared a common trunk with the DFA (5.0%), or originated from the FA distal to the DFA (1.7%, Table 2). There was no significant difference in the prevalence of origin between sides (p = 0.602); however, there was a significant difference between the sexes (p = 0.016). The MCFA originated from the DFA more commonly in males (50.0%) compared to females (13.3%). Origin from the FA proximal to the DFA was present with a significantly higher prevalence in females (16.7%), compared to males (13.3%). Origin from the FA distal to the DFA was present in only one limb on the left side of a male (1.7%). Furthermore, the MCFA shared a common trunk with the DFA on the left (3.3%) and right (1.7%) sides, and in both males (1.7%) and females (3.3%, Fig. 2).

**Table 2:** Prevalence of the variation in the origin of the medial circumflex femoral artery according to side and sex

Vessel of Origin	Total number of limbs (%)	Side		Sex	
		Left Number of limbs (%)	Right Number of limbs (%)	Male Number of limbs (%)	Female Number of limbs (%)
DFA	38 (63.3)	17 (28.3)	21 (35.0)	30 (50.0)	8 (13.3)
FA proximal to DFA	18 (30.0)	10 (16.7)	8 (13.3)	8 (13.3)	10 (16.7)
CT with DFA	3 (5.0)	2 (3.3)	1 (1.7)	1 (1.7)	2 (3.3)
FA distal to DFA	1 (1.7)	1 (1.7)	0 (0.0)	1 (1.7)	0 (0.0)
*p-value*		*0.602*		0.016*	

Key: *Significant; DFA- Deep Femoral Artery; FA- Femoral Artery; MCFA- Medial Circumflex Femoral Artery; CT- Common Trunk

**Fig. 3:**
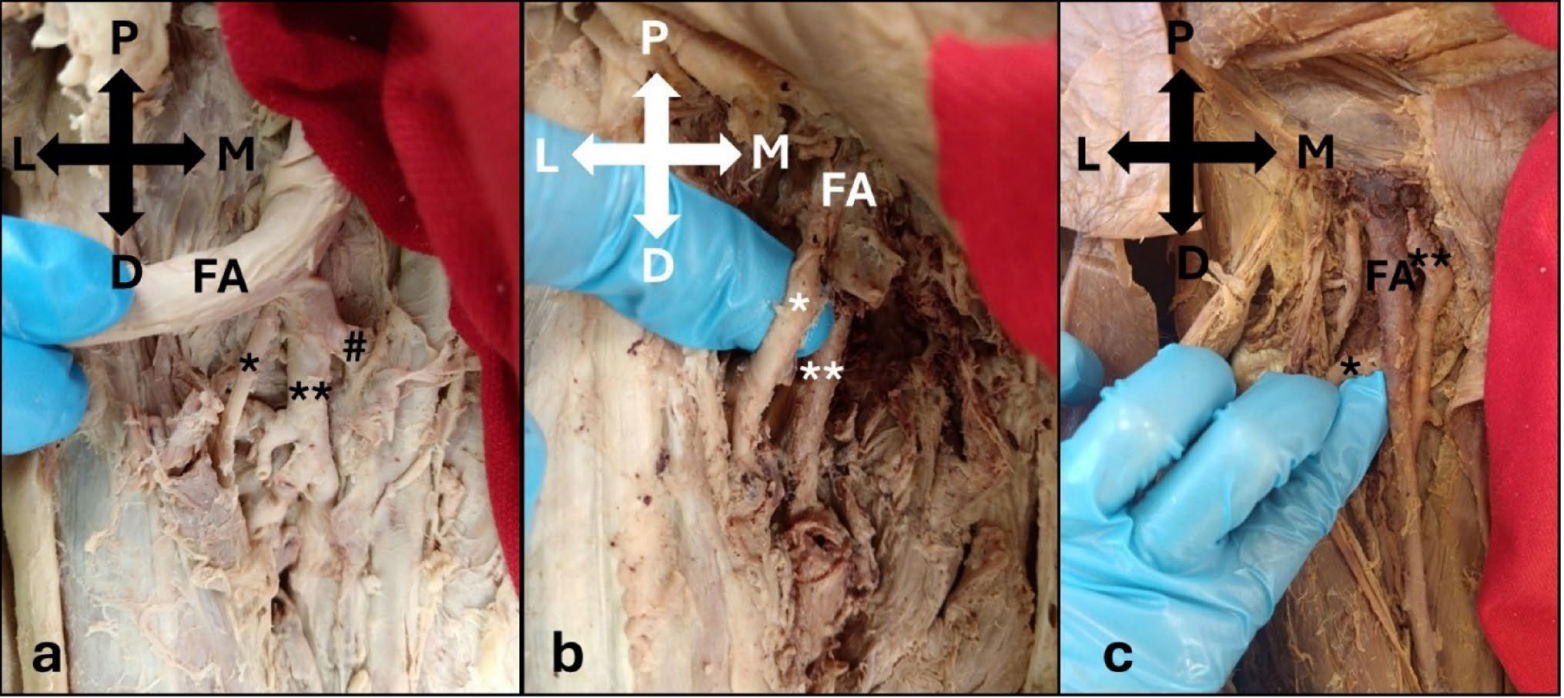
Photographs showing the variation in the origin of the lateral circumflex femoral artery (*), including the **a** origin from the deep femoral artery (**) (Right; Male); **b** origin from the femoral artery (FA) proximal to the deep femoral artery (**) (Right; Male; Femoral artery cut); and **c** origin from the FA distal to the deep femoral artery (**) (Right; Female). P, Proximal; D, Distal; L, Lateral; #, medial circumflex femoral artery

The LCFA originated from either the DFA (75.0%), shared a common trunk with the DFA (13.3%), the FA proximal to the DFA (10.0%), or the FA distal to the DFA (1.7%, Table 3). There were no significant differences between sides (p = 0.680); or sexes (p = 0.503). The LCFA on the left side (40.0%) originated more commonly from the DFA than on the right (35.0%), with a higher prevalence in males (50.0%) compared to females (25.0%). There was a slight right-sided predominance (6.7%) for the origin from the FA proximal to the DFA compared to the left side (3.3%), with males exhibiting a slightly higher prevalence of this origin (8.3%) than females (1.7%). The LCFA shared a common trunk with the DFA in 13.3% of lower limbs, with equal occurrences (6.7% respectively) present on both the left and right sides. This variation was observed in the lower limbs of both males (8.3%) and females (5.0%, Fig. 3).

**Table 3:** Prevalence of the variation in the origin of the lateral circumflex femoral artery according to side and sex

Vessel of Origin	Total number of limbs (%)	Side		Sex	
		Left Number of limbs (%)	Right Number of limbs (%)	Male Number of limbs (%)	Female Number of limbs (%)
DFA	45 (75.0)	24 (40.0)	21 (35.0)	30 (50.0)	15 (25.0)
CT with DFA	8 (13.3)	4 (6.7)	4 (6.7)	5 (8.3)	3 (5.0)
FA proximal to DFA	6 (10.0)	2 (3.3)	4 (6.7)	5 (8.3)	1 (1.7)
FA distal to DFA	1 (1.7)	0 (0.0)	1 (1.7)	0 (0.0)	1 (1.7)
*p-value*		*0.680*		*0.503*	

Key: DFA- Deep Femoral Artery; FA- Femoral Artery; LCFA- Lateral Circumflex Femoral Artery; CT- Common Trunk

The level of DFA origin varied between high (1-20 mm distal to MIP; 8.3%), middle (21-50 mm distal to MIP; 46.7%), and low (51-100 mm distal to MIP; 45.0%) origins (Table 4). There was no significant difference in the level of origin between sides (p = 0.627); however, there was a significant difference between the sexes (p = 0.031). High origins were observed more commonly in males (6.7%) than in females (1.7%). The middle origin was equally common in both males and females (23.3% respectively); however, a significant difference was present in low origins, where males had a considerably higher prevalence (36.7%) than females (8.3%).

**Table 4:** Variation in the level of the origin of the deep femoral artery according to side and sex

Level of DFA origin	Total number of limbs (%)	Side		Sex	
		Left Number of limbs (%)	Right Number of limbs (%)	Male Number of limbs (%)	Female Number of limbs (%)
High (1-20 mm distal to MIP)	5 (8.3)	3 (5.0)	2 (3.3)	4 (6.7)	1 (1.7)
Middle (21-50 mm distal to MIP)	28 (46.7)	12 (20.0)	16 (26.7)	14 (23.3)	14 (23.3)
Low (51-100 mm distal to MIP)	27 (45.0)	15 (25.0)	12 (20.0)	22 (36.7)	5 (8.3)
*p-value*		*0.627*		0.031*	

Key: *Significant, DFA- Deep Femoral Artery; MIP-Mid Point of the Inguinal Ligament

### Measurements

The inter- and intra-reliability tests confirmed that the methodology is reliable and replicable, with minimal error. The average differences fell within the established limits of agreement, showing no outliers and therefore strong concordance between the observers (Supplement 2; Figs. S4–S17).

The mean distance between the MIP and the DFA origin was 48.8 ± 12.7 mm, with no significant differences between sexes (p = 0.414; Table 5). The median distance between the origin of the MCFA and the origin of the DFA in males (10.6 ± 23.1 mm) and in females (4.3 ± 12.2 mm) was similarly statistically non-significant (p = 0.177). There was also no significant difference (p = 0.689) in the median distance between the origins of the LCFA and the DFA between males (8.1 ± 11.1 mm) and females (7.8 ± 13.8 mm). Furthermore, none of the distances were significantly different between left and right sides (Table 5), and none of the diameters of the femoral artery, deep femoral artery, medial circumflex lateral artery, or the lateral circumflex femoral artery were significantly different between sexes and sides (Table 6).

**Table 5:** Mean and median distances of the origins of the deep femoral artery and the medial and lateral circumflex femoral arteries according to side and sex

	Sides		Sex	
	Left	Right	Male	Female
Distance between the midpoint of the inguinal ligament and the origin of the deep femoral artery				
Mean ± Standard Deviation (range) (mm)	48.5 ± 12.3 (25.1- 73.9)	49.1 ± 13.3 (27.1- 77.8)	49.7 ± 12.5 (27.1- 72.0)	46.9 ± 13.2 (25.1- 77.8)
*p-value*	*0.756*		*0.414*	
Distance between the origin of the medial circumflex femoral artery and the origin of the deep femoral artery				
Median ± Interquartile Range (range) (mm)	7.5 ± 16.8 (0.0- 52.9)	7.5 ± 23.0 (0.0- 63.1)	10.6 ± 23.1 (0.0- 63.1)	4.3 ± 12.2 (0.0- 52.9)
*p-value*	*0.510*		*0.177*	
Distance between the origin of the lateral circumflex femoral artery and the origin of the deep femoral artery				
Median ± Interquartile Range (range) (mm)	7.9 ± 11.5 (0.0–30.4)	8.1 ± 13.8 (0.0–35.4)	8.1 ± 11.1 (0.0–35.4)	7.8 ± 13.8 (0.0–29.6)
*p-value*	*0.813*		*0.689*

**Table 6:** Mean diameters of the arteries according to side and sex

Vessel	Side Mean ± Standard Deviation ^/ Median ± Interquartile Range * (range) (mm)			Sex Mean ± Standard Deviation ^/Median ± Interquartile Range * (range) (mm)		
	Left	Right	*p-value*	Male	Female	*p-value*
Femoral artery	9.5 ± 1.4^ (5.6- 11.7)	9.2 ± 1.6^ (6.2- 12.3)	*0.118*	9.5 ± 1.6^ (5.6- 12.3)	9.1 ± 1.1^ (7.5–11.2)	*0.307*
Deep femoral artery	6.0 ± 1.0 * (3.7- 7.1)	5.8 ± 1.1 * (4.4- 8.3)	*0.393*	6.0 ± 0.8 ^ (3.7- 8.3)	5.8 ± 0.8 ^ (4.4- 7.5)	*0.531*
Medial circumflex femoral arteries	3.4 ± 0.3 * (2.8- 4.8)	3.6 ± 0.9^ (2.3- 6.0)	*0.910*	3.4 ± 0.7* (2.3- 6.0)	3.5 ± 0.7^ (2.4- 4.8)	*0.384*
Lateral circumflex femoral arteries	4.4 ± 0.6* (3.7- 6.1)	4.5 ± 0.8^ (3.6- 6.1)	*0.558*	4.6 ± 0.8^ (3.6- 6.1)	4.4 ± 0.6* (3.8- 6.1)	*0.259*

## Discussion

This study compared the variations of the FA, DFA, MCFA, and LCFA between sides of the body and sexes. Significant differences in the prevalence of variations in the origin of the MCFA and the level of DFA origin were found between the sexes, which could reflect sexual dimorphism. No significant differences were present in any of the measurements between sexes or sides. Variations of the DFA have been described by numerous authors from around the world; however, few studies have been done in South Africa (Supplement 3; Table S1). Furthermore, there is little published data of the external diameters of both the MCFA and LCFA from dissection studies. Variations in the DFA, MCFA, and LCFA are often congenital, and the variations observed in this study most likely resulted from the persistence and regression of intricate arterial networks formed during early embryonic development [8, 16].

A meta-analysis previously investigated the site of origin of the DFA from the FA and its distance from the midpoint of the inguinal ligament [27]. A total of 25 studies (2505 lower limbs) were included from Europe, Asia, North America and Africa, of which the majority were dissection-based. The findings were similar to that of the present study, with the DFA most commonly originating from the posterior aspect of the FA, although the pooled prevalence (38.8%) was lower than the prevalence of the present study (58.3%). The posterolateral origin was the second most prevalent in both the meta-analysis (34.0%) [27] and the present study (28.3%). However, after performing a sensitivity analysis including only the studies with a sample size larger than 100, the origins from the posterior, posterolateral and the lateral aspects were present with a similar prevalence. Most other studies have reported rates of the lateral origin as exceeding 15.0% [6, 22, 26]. However, Murthy et al. [15] documented no lateral origins in 70 lower limbs, while Manjappa et al. [13] reported a prevalence of 5.0% in 40 limbs, which is lower than the findings of the present study (10.0%). In contrast, higher incidences of posteromedial origins than the present study (1.7%) have been reported (20.0%, 8.0%, and 3.0%) [13, 15, 22]. Origin from the medial side appears to be rare, with a range of 0.0–3.1%, [15, 20, 22], within which the prevalence in the present study (1.7%) falls. Similarly, the authors of the meta-analysis concluded that the posteromedial, medial and anterolateral sites of origin are rare [27].

The DFA was the most common vessel of origin of the MCFA (63.3%), which is similar to previous reports ranging from 63.1 to 78.3% [2, 5, 7, 8]. This origin was significantly more prevalent in males (50.0%) than in females (13.3%), suggesting that variant origins are more common in females. However, as there were more males than females in this study, this finding should be interpreted with caution. Aligning with the present study (31.7%), origin from the FA ranges from 14.4% to 32.8% [5, 20, 28]. Similarly, origin from a common trunk with the DFA (5.0%) is within the published range of 1.6–15.0% [13, 16, 22, 30]. No other studies which investigated the origin of the MCFA reported significant differences between sexes or sides in the prevalence of variation [9, 16].

The LCFA most commonly originated from the DFA (75.0%), which is similar to previous studies according to a review (74.9%) [28]. A South African dissection study also reported this origin to be the most common in 78.9% of 90 bodies [14]. In comparison with Mogale et al. [14], the present study found a slightly higher prevalence of the LCFA origin from the DFA on the left (40.0% vs 37.8%) and a lower prevalence on the right (35.0% vs 41.1%). Origin from the FA was the second most prevalent in 11.7%, which is lower than Mogale et al. [14] (21.1%), but in agreement with the international literature (7.8–37.5%) [6, 7]. Additionally, the prevalence of a common trunk with the DFA (13.3%) is within the published range (1.6–16.2%) [6, 13, 22, 30]. While there were no significant differences in the prevalence of the LCFA origins between sexes, a previous study reported variant LCFA origin as more common in females [16].

The most prevalent (46.7%) level of origin was the middle (21–50 mm), which is similar to a previous study (41.1%) [5]. The low origin was the second most common (45.0%); and the high origin the least common (8.3%), whereas in contrast, Claassen et al. [5] reported the low origin as the least common (19.6%). Both lower and higher levels of origin were more common in males (36.7% and 50.0%, respectively), and similarly, Claassen et al. [5] reported the low origin as more prevalent in males (13.1%) than in females (6.5%). These results suggest that males may be more likely to exhibit a lower origin of the DFA than females, and healthcare providers may consider modifying their approach to vascular access according to the biological sex of the patient. The difference in the level of origin of the DFA may reflect sexual dimorphism as males tend to be taller than females.

The mean distance between the MIP and the DFA (48.8 ± 12.7 mm) falls within the range of published means (36.0–51.5 mm) [15, 16, 27]. Although not significant between sexes in the present study, the previously described meta-analysis reported the distance as significantly longer in males (53.5 mm) than in females (48.8 mm) [27]. The distances on the right (48. ± 12.3 mm) and left (49.1 ± 13.3 mm) in the present study are smaller than the pooled mean distances (55.9 mm and 59.4 mm, respectively), and did not differ significantly, while a significant difference was present between sides in the meta-analysis [27]. Other studies did not find any significant differences between sides [15, 16] and sexes [16].

The median distance between the origins of the MCFA and the DFA **(**7.5 ± 20.9 mm) is smaller than the published range (23.0–48.5 mm) [13, 15, 16, 20], whereas the median distance between the origins of the LCFA and the DFA (8.0 ± 12.4 mm) is within the reported range (2.0–25.0 mm [12, 15, 16, 20]. The median distance of the MCFA in males (10.6 mm) and females (4.3 mm) are smaller than those reported by Nasr et al. (19.1 mm and 18.9 mm, respectively) [16]. Similar to the present study, no significant differences in the distances of both the MCFA and the LCFA have been reported between sexes [16] and sides [15, 16]. The difference in findings between studies could be due to variations in sample size, demographic differences, or differences in methodological approaches.

The mean diameters of the FA (9.4 ± 1.5 mm) and DFA (5.9 ± 0.8 mm) fall within the previously published ranges ((4.0–9.6 mm) [8, 12, 15, 17] and (2.5–7.31 mm) [4, 8, 12, 15, 17], respectively). Similarly, the diameters of the MCFA and LCFA are similar to those reported by previous research [8, 12]. Although not statistically significant in the present study, other studies have reported differences between the sexes, with diameters generally larger in males [8, 17]. There were no significant differences in any of the diameters between sides in the present or previous studies [8, 15].

Recognising and understanding the variations in the origin and location of the DFA, MCFA, and LCFA are crucial for orthopaedic, plastic reconstructive, vascular and interventional radiological procedures [28]. A high origin of the DFA may increase the risk of complication during hip joint access procedures, as demonstrated by a case of iatrogenic injury following intertrochanteric hip fracture surgery [28]. Furthermore, vascular injury to the DFA may lead to the formation of pseudoaneurysms or arteriovenous fistulas [23]. The presence of a high origin of the DFA should be investigated prior to performing femoral artery access and nerve blocks in the femoral triangle, as its presence may increase the risk of iatrogenic vascular injury [28]. The LCFA is vital for supplying anterolateral thigh flaps, and neglecting to consider variations of this vessel may jeopardize reconstructive efforts [28]. Finally, awareness of MCFA variations is particularly important to prevent avascular necrosis of the femoral head during osteotomies [13].

Knowledge of the range of diameters of the FA, DFA, MCFA, and LCFA is crucial for surgical planning and interventions, as understanding these measurements allows clinicians to anticipate the sizes of vessels they will encounter during procedures [19]. Diameters may indicate vascular health and integrity, with deviations from the accepted standard ranges of these vessels suggesting pathological conditions such as aneurysms or other vascular diseases, which may complicate surgical outcomes or require intervention. In addition, vessel diameter influences blood flow dynamics and resistance within the arterial system, while the presence of plaques may reduce blood flow and increase the measured external diameter; thus knowledge of the range of diameters is important for hemodynamic studies [19].

This study faced several limitations, including the availability of body donors, which resulted in unequal representation of male and female donors. No medical history or biometric data (e.g., height and weight) was available, preventing adjustments for size differences or vascular conditions that might have influenced measurements. Although the study was conducted over 10 months, only three months were allocated for data collection, and as some bodies were excluded due to damage from previous dissection, the sample size is relatively small. As the body donors were embalmed, the measurements may not represent those of living individuals. In addition, the branches of the MCFA and the LCFA were not investigated and it is possible that variations in the origin of these vessels were present.

## Conclusion

This study found variation in the origin, location and range of dimensions of the DFA, MCFA and LCFA. Significant differences were found between sexes in the prevalence of variation in the origin of the MCFA and the level of DFA origin. No significant differences were present between sides and sexes regarding distances or diameters. Recognition of these variations is crucial for clinicians undertaking procedures in the femoral triangle and may reduce the risk of iatrogenic injury to these vessels. Future research should aim to include equal numbers of males and females in ultrasound based studies, which may overcome some of the limitations of an embalmed body donor sample. Future studies could investigate the branches of the LCFA and MCFA as these are also known to vary.

## Supplementary Information

Below is the link to the electronic supplementary material.

Supplementary Material 1

## Data Availability

On request.
