# Supplementary material for: Anatomical variations of the deep femoral artery: a dissection-based study
Source: Surg Radiol Anat. 2025 Sep 15;47(1):205. doi: 10.1007/s00276-025-03702-z (PMC12436538; doi:10.1007/s00276-025-03702-z)
Supplement: Supplementary file 1 — Supplementary Material 1 [file 276_2025_3702_MOESM1_ESM.docx]

# **Supplementary Material**

##
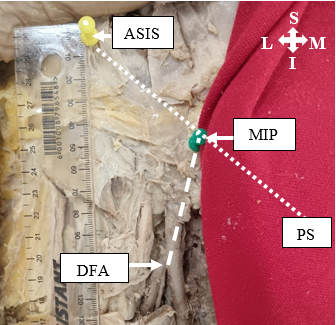
Supplement 1

**Fig S1** Photograph showing the method for measuring the distance (dashed line) between the midpoint of the inguinal ligament (MIP) and the origin of the deep femoral artery (DFA; Left; Female). Pins were placed at the anterior superior iliac spine (ASIS) and the pubic symphysis (PS; under the cloth). The midpoint (dotted line) between these two markers was subsequently determined, and an additional pin was positioned at the midpoint as a reference point for measurement. An ORIGIN digital calliper (0-150 mm) was utilised to measure the distance from the MIP to the origin of the DFA.

**
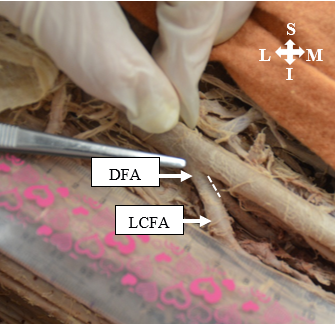
**

**L**

**I**

**M**

**S**

**Fig S2** Photograph showing the method for measuring the distance (dashed line) between the origin of the deep femoral artery (DFA) and the origin of the lateral circumflex femoral artery (LCFA; Right, Female). An ORIGIN digital calliper (0-150 mm) was used to measure the distance. A similar method was used to measure the distance between the origin of the DFA and the origin of the medial circumflex femoral artery.

**
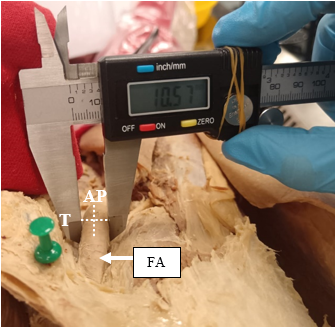
Fig S3** Photograph showing the method for measuring the external diameter of the femoral artery (FA) above its bifurcation point. Anteroposterior (AP) and transverse (T) distances were measured (dashed lines). The same methods were repeated for measurements of the external diameter of the deep femoral artery, medial circumflex femoral artery and lateral circumflex femoral artery, at their respective points of origin.

## Supplement 2

**Inter-Observer Reliability Bland-Altman Graphs**

**Fig S4** Bland-Altman plot demonstrating the inter-observer error for the measurements taken of the distance between the midpoint of the inguinal ligament and the origin of the deep femoral artery (95% confidence interval = 9.3 mm)

**Fig S5** Bland-Altman plot demonstrating the inter-observer error for the measurements taken of the distance between the origin of the deep femoral artery and the origin of the medial circumflex femoral artery (95% confidence interval = 1.5 mm)

**Fig S6** Bland-Altman plot demonstrating the inter-observer error for the measurements taken of the distance between the origin of the deep femoral artery and the origin of the lateral circumflex femoral artery (95% confidence interval = 2.4 mm)

**Fig S7** Bland-Altman plot demonstrating the inter-observer error for the measurements taken of the diameter of the femoral artery (95% confidence interval = 2.8 mm)

**Fig S8** Bland-Altman plot demonstrating the inter-observer error for the measurements taken of the diameter of the deep femoral artery (95% confidence interval = 2.5 mm)

**Fig S9** Bland-Altman plot demonstrating the inter-observer error for the measurements taken of the diameter of the medial circumflex femoral artery (95% confidence interval = 2.2 mm)

**Fig S10** Bland-Altman plot demonstrating the inter-observer error for the measurements taken of the diameter of the lateral circumflex femoral artery (95% confidence interval = 2.7 mm)

**Intra-Observer Reliability Bland-Altman Graphs**

**Fig S11** Bland-Altman plot demonstrating the intra-observer error for the measurements taken of the distance between the midpoint of the inguinal ligament and the origin of the deep femoral artery (95% confidence interval = 7.3 mm)

**Fig S12** Bland-Altman plot demonstrating the intra-observer error for the measurements taken of the distance between the origin of the deep femoral artery and the origin of the medial circumflex femoral artery (95% confidence interval = 2.9 mm)

**Fig S13** Bland-Altman plot demonstrating the intra-observer error for the measurements taken of the distance between the origin of the deep femoral artery and the origin of the lateral circumflex femoral artery (95% confidence interval = 1.5 mm)

**Fig S14** Bland-Altman plot demonstrating the intra-observer error for the measurements taken of the diameter of the femoral artery (95% confidence interval = 1.5 mm)

**Fig S15** Bland-Altman plot demonstrating the intra-observer error for the measurements taken of the diameter of the deep femoral artery (95% confidence interval = 1.4 mm)

**Fig S16** Bland-Altman plot demonstrating the intra-observer error for the measurements taken of the diameter of the medial circumflex femoral artery (95% confidence interval = 1.1 mm)

**Fig S17** Bland-Altman plot demonstrating the intra-observer error for the measurements taken of the diameter of the lateral circumflex femoral artery (95% confidence interval = 1.3 mm)

## Supplement 3

**Table S1** Comparative Overview of Study Characteristics

| Author | Country | Type of Study | Number of lower limbs |
| --- | --- | --- | --- |
| Present Study | South Africa | Anatomical Dissection | 60 |
| Lunn-Collier et al. (2024) [11] | South Africa | Computed Tomography Angiography | 224 |
| Murthy et al. (2022) [15] | India | Anatomical Dissection | 70 |
| Patel et al. (2022) [17] | USA | Anatomical Dissection | 155 |
| Mogale et al. (2021) [14] | South Africa | Anatomical Dissection | 90 |
| Claassen et al. (2021) [5] | Germany | Anatomical Dissection | 111 |
| Ma et al. (2021) [12] | China | Anatomical Dissection | 115 |
| Kaur et al. (2019) [7] | India | Anatomical Dissection | 40 |
| Łabętowicz et al. (2019) [8] | Poland | Anatomical Dissection | 80 |
| Vuksanović-Božarić et al, (2018) [29] | Montenegro | Microdissection | 60 |
| Rajani et al. (2015) [21] | India | Anatomical Dissection | 33 |
| Aghera et al. (2015) [1] | India | Anatomical Dissection | 102 |
| Chauhan et al. (2015) [4] | India | Anatomical Dissection | 51 |
| Manjappa et al. (2014) [13] | India | Anatomical Dissection | 80 |
| Anjankar et al. (2014) [2] | India | Anatomical Dissection | 120 |
| Nasr et al. (2014) [16] | Saudi Arabia | Anatomical Dissection | 90 |
| Dixit et al. (2011) [6] | India | Anatomical Dissection | 228 |
| Prakash et al. (2010) [20] | India | Anatomical Dissection | 64 |
| Üzel et al. (2008) [28] | Turkey | Anatomical Dissection | 110 |
